# Distinct Responses of Abundant and Rare Soil Bacteria to Nitrogen Addition in Tropical Forest Soils

**DOI:** 10.1128/spectrum.03003-22

**Published:** 2023-01-09

**Authors:** Jinhong He, Xiangping Tan, Yanxia Nie, Lei Ma, Juxiu Liu, Xiankai Lu, Jiangming Mo, Julie Leloup, Naoise Nunan, Qing Ye, Weijun Shen

**Affiliations:** a Key Laboratory of Vegetation Restoration and Management of Degraded Ecosystems, South China Botanical Garden, Chinese Academy of Sciences, Guangzhou, China; b Key Laboratory of Geospatial Technology for Middle and Lower Yellow River Regions (Henan University), Ministry of Education, Kaifeng, China; c Institute of Ecology and Environmental Sciences–Paris, Sorbonne Université CNRS-IRD-INRAE-Université Paris Cité-UPEC, Paris, France; d Department of Soil and Environment, Swedish University of Agricultural Sciences, Uppsala, Sweden; e College of Life Sciences, Gannan Normal University, Ganzhou, China; f Guangxi Key Laboratory of Forest Ecology and Conservation, State Key Laboratory for Conservation and Utilization of Agro-bioresources, College of Forestry, Guangxi University, Nanning, Guangxi, China; University of Mississippi

**Keywords:** abundant and rare taxa, bacterial community, community assembly, nitrogen addition, tropical forest

## Abstract

Soil microbial responses to anthropogenic nitrogen (N) enrichment at the overall community level has been extensively studied. However, the responses of community dynamics and assembly processes of the abundant versus rare bacterial taxa to N enrichment have rarely been assessed. Here, we present a study in which the effects of short- (2 years) and long-term (13 years) N additions to two nearby tropical forest sites on abundant and rare soil bacterial community composition and assembly were documented. The N addition, particularly in the long-term experiment, significantly decreased the bacterial α-diversity and shifted the community composition toward copiotrophic and N-sensitive species. The α-diversity and community composition of the rare taxa were more affected, and they were more closely clustered phylogenetically under N addition compared to the abundant taxa, suggesting the community assembly of the rare taxa was more governed by deterministic processes (e.g., environmental filtering). In contrast, the abundant taxa exhibited higher community abundance, broader environmental thresholds, and stronger phylogenetic signals under environmental changes than the rare taxa. Overall, these findings illustrate that the abundant and rare bacterial taxa respond distinctly to N addition in tropical forests, with higher sensitivity of the rare taxa, but potentially broader environmental acclimation of the abundant taxa.

**IMPORTANCE** Atmospheric nitrogen (N) deposition is a worldwide environmental problem and threatens biodiversity and ecosystem functioning. Understanding the responses of community dynamics and assembly processes of abundant and rare soil bacterial taxa to anthropogenic N enrichment is vital for the management of N-polluted forest soils. Our sequence-based data revealed distinct responses in bacterial diversity, community composition, environmental acclimation, and assembly processes between abundant and rare taxa under N-addition soils in tropical forests. These findings provide new insight into the formation and maintenance of bacterial diversity and offer a way to better predict bacterial responses to the ongoing atmospheric N deposition in tropical forests.

## INTRODUCTION

As one of the major global change drivers, atmospheric nitrogen (N) deposition has dramatically increased over the past few decades with industrialization and urbanization and is expected to increase further in the future (1). The excessive loading of anthropogenic reactive N can impact plant primary production, biodiversity and cause soil eutrophication, acidification and element-cycling imbalances (2–4), all of which can consequently influence ecosystem structure and services (5, 6). Soil bacterial communities constitute a large part of belowground biodiversity (7, 8) and play pivotal roles in multiple ecosystem functions (9, 10), such as litter decomposition, nutrient cycling, and climate regulation (11, 12). Atmospheric N deposition has been found to have profound effects on soil bacterial diversity, composition, activity, and function, directly through its effect on microbial nutrition and indirectly through the alteration of edaphic conditions (13–15). For instance, N inputs can decrease soil bacterial diversity, due to the extinction of some taxa that are adapted to nutrient-poor conditions or acidic intolerant species, following N-induced soil acidification (16, 17). Such changes may also result in alterations of microbial community composition, activities, interactions, and their contributions to ecosystem function (18–20). Therefore, a deeper understanding of soil bacterial community responses to N enrichment would give a finer understanding of the potential consequences of N deposition for terrestrial ecosystem functioning (21, 22).

Historically, the responses of soil bacterial communities to N additions have been extensively studied at the overall community level. However, soil bacterial communities are highly diverse and complex and usually present strongly skewed relative abundance distributions, with a few abundant or common species and a large number of rare species (23, 24). Previous studies have mostly focused on the abundant members, due to the belief that their contributions to biogeochemical cycling are the most significant, whereas the rare species have usually been neglected (23). In fact, the rare species are ecologically important, as the rare taxa possess great genetic diversity and substantial metabolically active lineages (23, 25). Additionally, the rare taxa serve as a “seed bank” that can proliferate under appropriate conditions, maintaining bacterial diversity and composition and thus contributing to ecosystem functioning (26, 27). The rare bacterial taxa have been found to play essential roles in regulating soil fertility (28), phosphorus mineralization (29), sulfate reduction (30), multinutrient cycling (31, 32), and maintenance of community diversity (33). Moreover, the abundant and rare bacterial taxa have been demonstrated to have disparate functional characteristics, activities, and ecological strategies. For instance, the abundant taxa have wider niche breadth, higher competitive ability, and broader environmental acclimation than the rare taxa (24, 25, 34). Therefore, the abundant and rare bacterial taxa generally exhibit distinct responses to anthropogenic or natural environmental perturbations, including wetting-drying cycles (35), pyrene stress (36), fertilization (21), oil contamination (37), and vegetation restoration (38) in agricultural and desert ecosystems. Furthermore, the rare taxa are more responsive to environmental perturbations than the abundant taxa. Such differentiated responses of the abundant and rare bacterial taxa could potentially affect their roles in community processes and functioning (39, 40). However, the responses of community structure and dynamics of the abundant and rare bacterial taxa to N addition in forest ecosystems, which are crucial for predicting and elucidating ecosystem responses in the context of elevated anthropogenic N deposition, are still unknown.

Microbial community assembly is mainly governed by deterministic (e.g., selection via environmental filtering and biotic interactions) and stochastic (e.g., dispersal process, ecological drift, and diversification) processes (41, 42), and so are the abundant- and rare-taxon communities, but the relative importance of each process remains controversial (39, 43). For instance, rare bacterial taxa were found to be primarily governed by deterministic processes in coastal wetland, agricultural, and garden soils (43–45), while opposing results had been observed in grassland and oil-contaminated soils (39, 46). These observed differences may be ascribed to the different geographic scales, habitat conditions, and/or diverse environmental perturbations (47–49). Nevertheless, studies assessing the community assembly of abundant and rare bacterial taxa in forest soils under anthropogenic N enrichment remain scarce, though stochastic processes have been found to control the assembly of the soil microbial community at the overall community level under global N deposition scenarios (18).

Tropical and subtropical forests, which play critical roles in regulating nutrient and hydrological cycles (50), are suffering from increasing N deposition (51). Here, we made use of a short-term (~2 years) and long-term (~13 years) (52) N addition experiments to explore how abundant and rare bacterial taxa respond to exogenous N inputs. Specifically, our objectives were to (i) uncover the response patterns of the abundant and rare bacterial taxa by highlighting their susceptibility to anthropogenic N inputs and (ii) identify the factors and mechanisms that structure the assembly processes of the abundant and rare communities. Given that rare bacterial taxa had higher metabolic activity, niche specialization, lower competitive ability, and environmental adaptability (25, 37, 43), we hypothesized that (i) N additions would affect the diversity and community composition of the rare bacterial taxa more significantly than those of the abundant taxa and (ii) the community assembly of the rare taxa is mainly driven by deterministic processes, whereas that of the abundant taxa is instead mainly governed by stochastic processes.

## RESULTS

### Distributions and α-diversity of the abundant and rare bacterial taxa under N addition.

Overall, 434,885 and 434,910 high-quality sequences and 5,450 and 5,529 amplicon sequence variants (ASVs) were obtained from the short- and long-term N treatment samples, respectively. A high proportion of the ASVs (91.0% and 91.1% in the short- and long-term experiments, respectively), contributing 31.4% and 31.7% of short- and long-term sequences, were attributed to rare taxa. A low proportion of the ASVs (9.1% and 8.9% in the short- and long-term experiments, respectively), containing 68.6% and 68.3% of short- and long-term sequences, were classified as abundant taxa (see Table S1 in the supplemental material). Venn diagrams showed that almost all of the ASVs classified as abundant taxa were present in all samples, whereas many of the rare ASVs were found only in individual or several N treatments of the short- and long-term experiments (Fig. S1).

**FIG 1:**
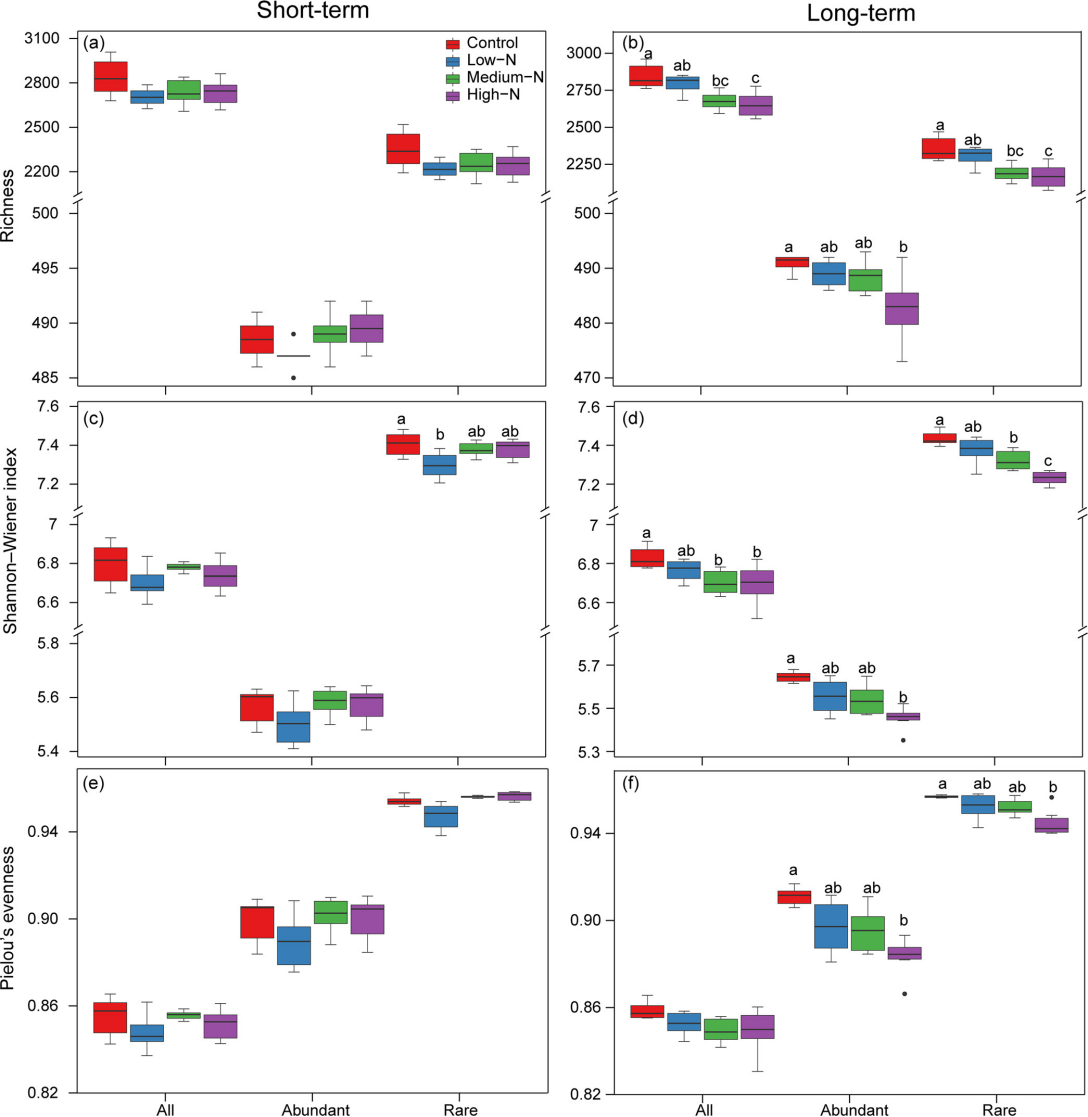
(a to f) Variations of α-diversity indices of the overall, abundant, and rare taxa across N addition levels in the short- (a, c, and e) and long-term (b, d, and f) experiments. The different lowercase letters (a, b, c) denote the significances (*P < *0.05) of α-diversity among N addition levels in each experiment.

No clear trends in diversity indices were observed with N additions in the short-term experiment: only the Shannon index of the rare taxa in the low-N treatment was significantly different (*P < *0.05). In the long-term experiment, there were clear trends in all the α-diversity indices (richness, Shannon index, and Pielou’s evenness) of the abundant and rare taxa, where significant decreases with the increasing levels of N were observed (*P < *0.05; Fig. 1). Additionally, the α-diversity indices of the rare taxa were higher and more variable than those of the abundant taxa in the long-term experiment.

### Changes in the composition of the abundant and rare bacterial taxa in response to N addition.

The vast majority of the sequences (97.6%) and ASVs (92.6%) belonged to eight major phyla in the short-term experiment (Fig. S2). Among them, 68.2% of sequences and 17.5% of ASVs were abundant taxa, whereas 29.4% of sequences and 75.1% of ASVs were assigned to rare taxa. Generally, *Acidobacteria* (47.2%, 37.1%, and 10.1% of overall, abundant, and rare taxa, respectively) was the most dominant phylum, followed by *Proteobacteria* (27.1%, 17.4%, and 9.7% of overall, abundant, and rare taxa, respectively). Similarly, 97.7%, 67.8%, and 29.9% of sequences and 93.0%, 17.6%, and 75.4% of ASVs of overall, abundant, and rare taxa can be assigned to eight major phyla in the long-term experiment.

**FIG 2:**
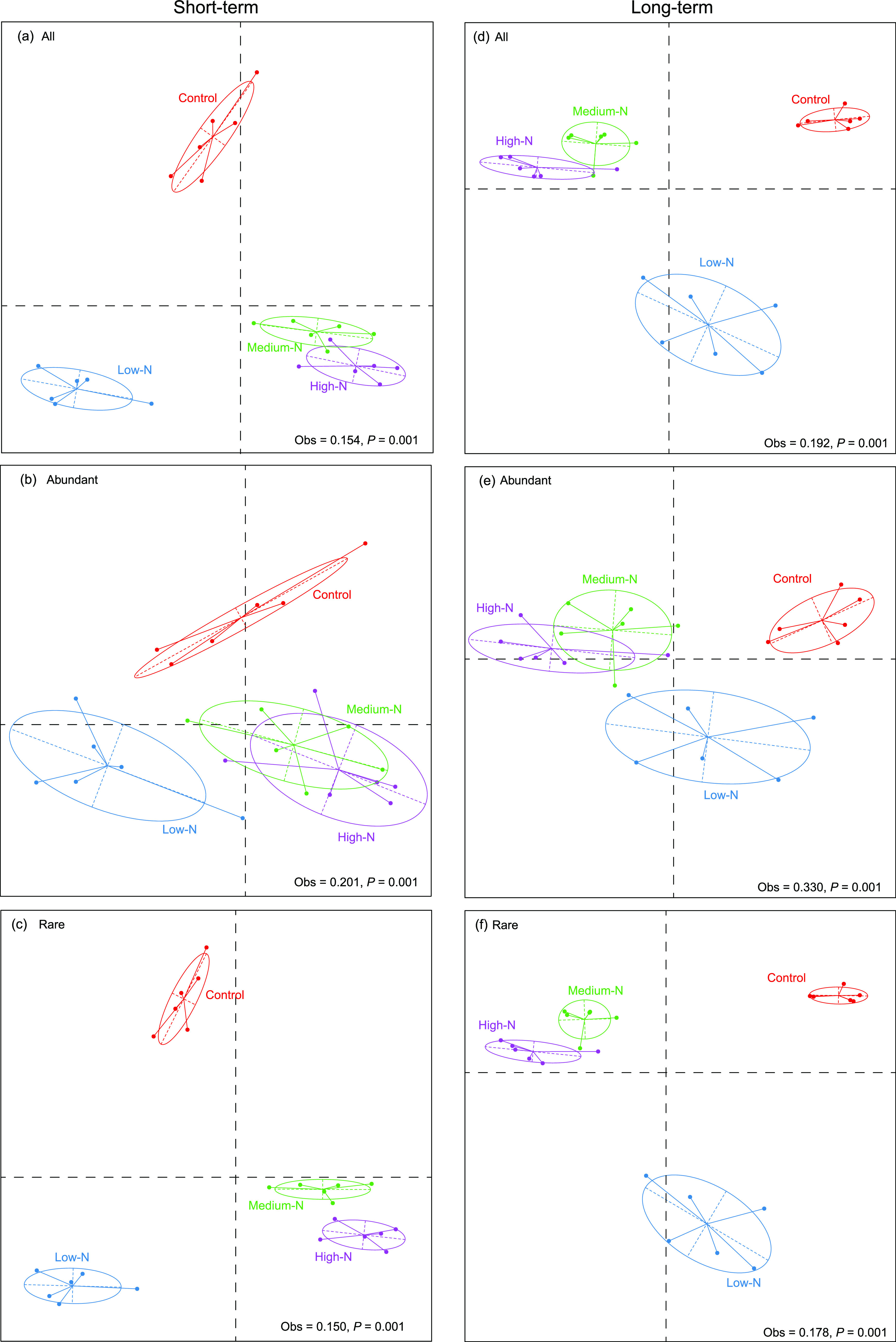
(a to f) Between-class analysis (BCA) of bacterial community composition of the overall, abundant, and rare taxa among the four N addition treatments in the short- and long-term experiments, respectively. All the BCA ordinations are significant (*P < *0.05).

The effects of N addition treatments on the community compositions of overall, abundant, and rare bacterial taxa in the short- and long-term experiments are presented in Fig. 2. A significant effect of N addition (*P < *0.05) was observed between control and N addition levels (control versus low N, medium N, and high N) in the two experiments, highlighting the fact that N addition shifted the composition of all bacterial communities. Moreover, a clear distinction among community compositions of the overall and rare taxa across the four treatments was observed in the two experiments, whereas the abundant bacterial composition did not form distinct clusters between medium- and high-N treatments (Fig. 2b and e).

**FIG 3:**
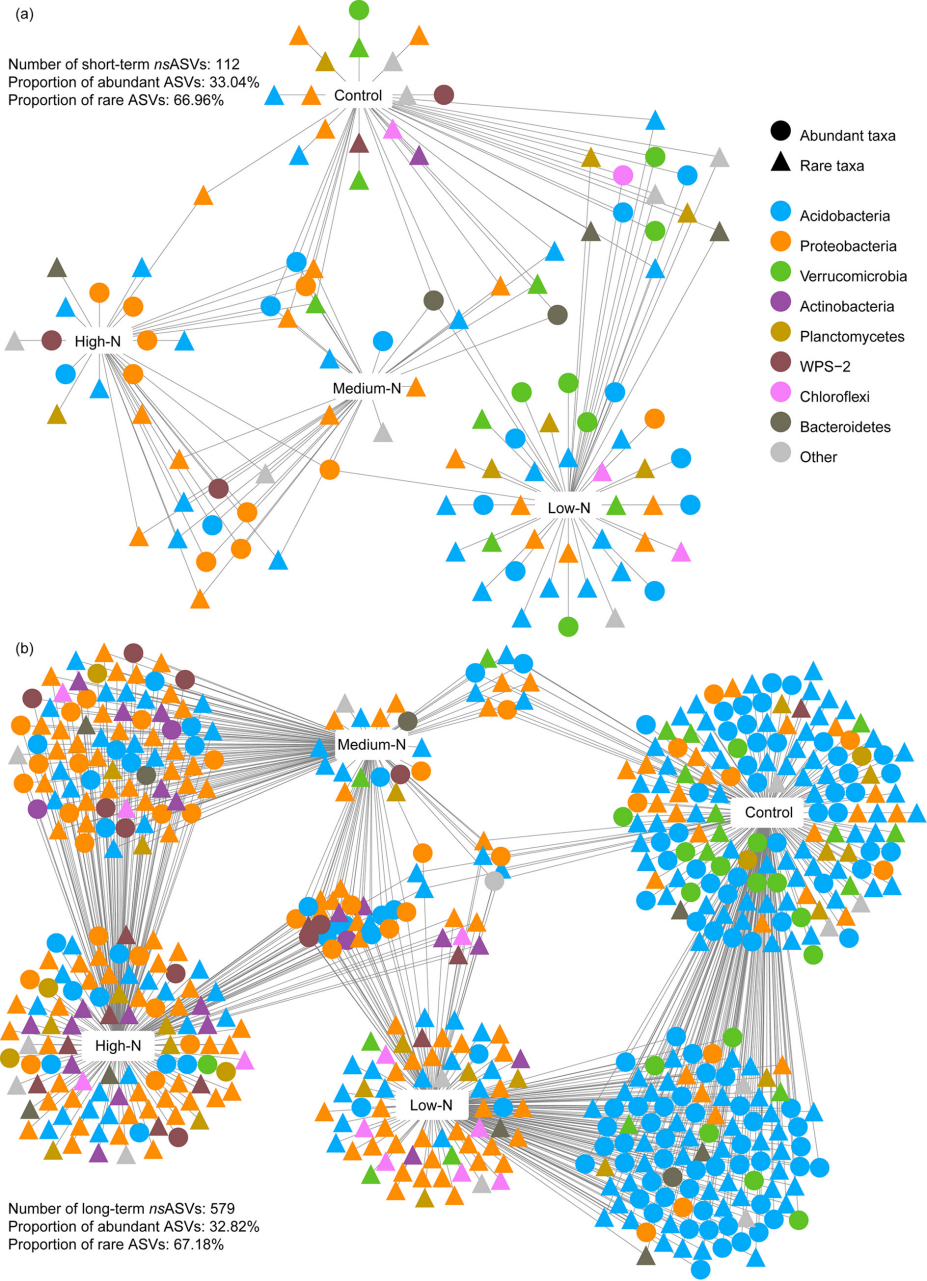
(a and b) Bipartite networks depict the associations between the bacterial nsASVs and the different N addition levels (estimated via the indicator species analysis in the indicspecies package and likelihood ratio tests with the edgeR package) in the short- (a) and long-term (b) experiments. Nodes represent ASVs positively and significantly associated (*P < *0.05) with one or more N addition levels. ASVs are colored according to their phylum assignment. Edges represent the associations of individual ASVs with different N levels.

More N-sensitive ASVs (nsASVs; *P < *0.05) were found in the long-term experiment than in the short-term experiment (Fig. 3a and b). The majority of nsASVs (~67%) were rare taxa, whereas about 33% of the nsASVs were abundant taxa in both experiments. The association strengths (correlation coefficient, *r*) of ASVs ranged from 0.4 to 0.8 and 0.4 to 0.9 in the short- and long-term experiments, respectively. Specifically, 65.2% and 58.9% of nsASVs were associated with individual treatments in the short- and long-term experiments, respectively, resulting in distinct bacterial communities in the four N levels. Approximately 34.8% and 41.1% of nsASVs were associated with two or three treatments in the short- and long-term experiments, respectively. Additionally, more than 57.1% of sensitive rare taxa were associated with the single treatment in both experiments. *Acidobacteria* were mainly enriched in control (63.7%) and cross-combinations of control and low-N (94.3%), while *Proteobacteria* dominated under high N (41.6%) and cross-combinations of medium N and high N (48.2%) in the long-term experiment (Fig. 3b). Moreover, the relative abundance and relative frequencies of sensitive *Acidobacteria* and *Verrucomicrobia* were decreased with increasing N addition, whereas those of *Proteobacteria*, *Actinobacteria*, *Planctomycetes*, and WPS-2 were enhanced by N addition (especially the high N) in the long-term experiment (*P < *0.05; Fig. S3c and d). However, no such distinct pattern was found among the four N levels in the short-term experiment (Fig. S3a and b).

### Environmental responses of the abundant and rare bacterial taxa.

The α-diversity of overall and abundant taxa was positively correlated with total organic carbon (TOC) and the total organic carbon/total phosphorus ratio (C/P) and negatively correlated with soil pH and dissolved organic carbon (DOC) in the short-term experiment. Soil pH was positively correlated with bacterial α-diversity of overall, abundant, and rare taxa in the long-term experiment (*P < *0.05; Table S2). The results of simple Mantel tests demonstrated that C/P and pH were positively correlated with the bacterial community composition of overall, abundant, and rare taxa in the short- and long-term experiments, respectively (*P < *0.05; Table S3). The relative abundance of the major phyla of overall, abundant, and rare taxa was mainly correlated with total nitrogen/total phosphorus ratio (N/P) and C/P in the short-term experiment, while the relative abundance mainly correlated with soil pH in the long-term experiment (*P < *0.05; Fig. S4).

**FIG 4:**
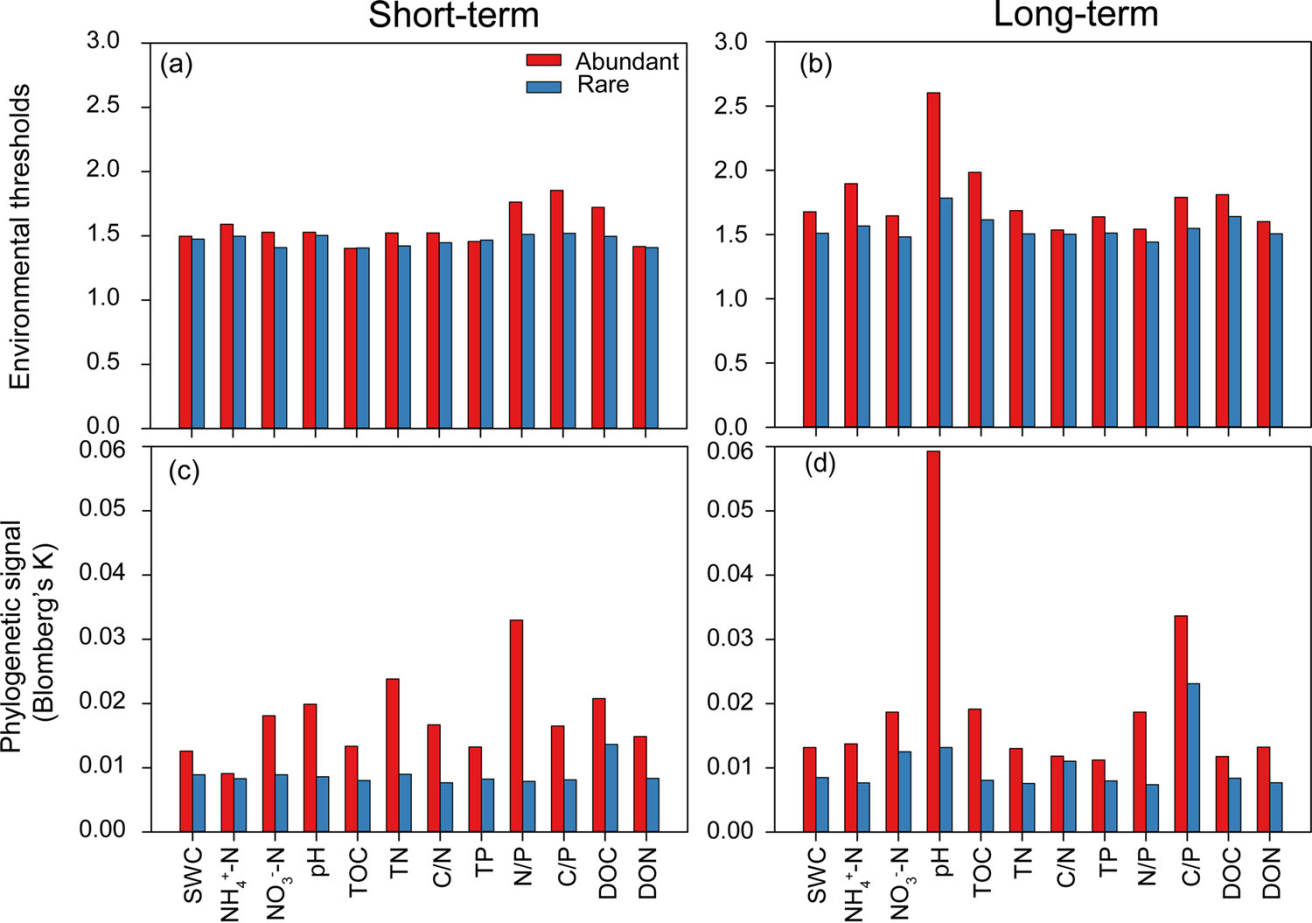
Environmental acclimation of the abundant and rare bacteria in the short- and long-term N addition soils. (a and b) Environmental breadths assessed by the threshold values of the abundant and rare taxa in response to environmental variables determined applying threshold indicator taxon analyses (TITAN). (c and d) Phylogenetic signals of the abundant and rare taxa reflecting the trait conservatism for environmental preferences employing Blomberg’s *K* statistic. The abbreviations of environmental variables are defined in Materials and Methods.

The abundant bacterial taxa exhibited greater environmental breadth than the rare taxa for all soil physicochemical variables in both experiments, with most environmental thresholds being higher for abundant taxa in the long- than the short-term experiments (Fig. 4a and b). Blomberg’s *K* statistic revealed that the abundant taxa exhibited stronger phylogenetic signals for all environmental variables compared with the corresponding rare taxa in both experiments (Fig. 4c and d). Additionally, the phylogenetic signals were stronger for soil pH and C/P in the long- than the short-term experiment within abundant and rare taxa, respectively.

### Ecological community assembly processes of the abundant and rare bacterial taxa.

**FIG 5:**
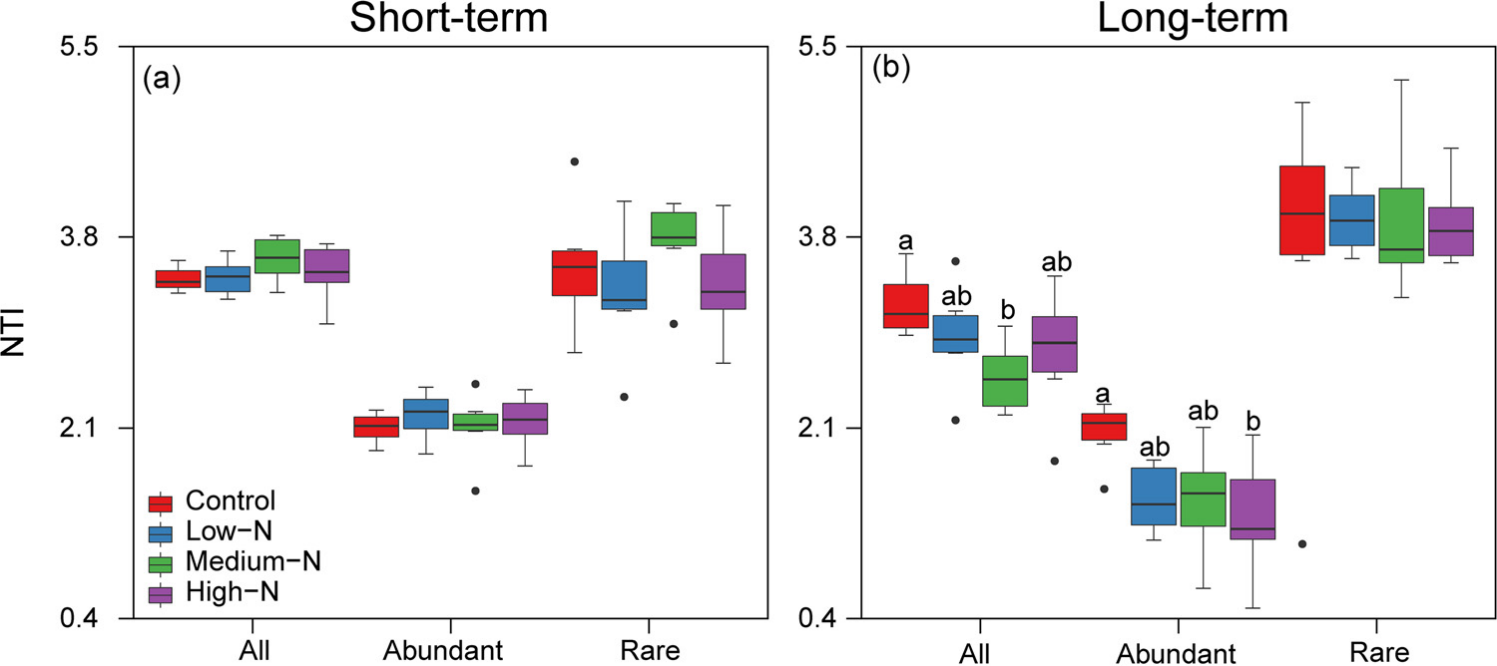
(a and b) Mean nearest taxon index (NTI) of overall, abundant, and rare bacterial taxa across N addition levels in the short- (a) and long-term (b) experiments. The different lowercase letters (a, b, c) denote the significances (*P < *0.05) of NTI values among N addition levels in each experiment.

The mean nearest taxon index (NTI) values were larger than zero for overall, abundant, and rare taxa in the short- and long-term experiments (Fig. 5), indicating that these communities were more closely related to themselves than expected by chance (i.e., phylogenetic clustering) across all treatments in both experiments, thus suggesting the dominance of deterministic processes in their community assembly. However, the NTI values of the abundant taxa were significantly lower than those of the rare taxa (*P < *0.05), suggesting relatively higher contributions of deterministic processes in the community assembly of the rare taxa than that of the abundant taxa. Additionally, N addition significantly decreased the NTI values of overall and abundant taxa in the long-term experiment, but no significant effects of N addition on NTI values were found for the three taxon groups in the short-term experiment (Fig. 5).

## DISCUSSION

### Dynamics of abundant and rare bacterial taxa under N addition.

In the present study, we found that N addition (especially in the long-term experiment) significantly decreased the bacterial α-diversity (Fig. 1), which is in line with previous studies of forest ecosystems (16, 17). One possible explanation is that excessive N input results in intense competition of soil bacteria for other resources (e.g., carbon resources; 53) and the exclusion of less competitive rare species, thereby reducing the diversity. The decreased soil pH would also contribute to the results, as pH can affect the membrane-bound proton pumps and protein stability, resulting in relatively narrow growth tolerances of bacteria to pH (54, 55). Moreover, N additions can increase the abundance of soil bacterivores (56), which may further reduce bacterial diversity due to top-down effects. Additionally, the greater decrease of bacterial α-diversity in the long-term experiment than the short-term experiment might be attributed to greater changes in soil microhabitats (e.g., soil pH) under long-term N addition.

Nevertheless, the α-diversity and community composition of the rare taxa were more influenced by N addition than those of the abundant taxa (Fig. 1 and 2), supporting our first hypothesis. The results highlight the fact that the abundant taxa possess higher potential resilience or resistance, whereas the rare taxa are more sensitive to the disturbances (e.g., N addition). One plausible explanation is that the abundant taxa occupy wider niches, have higher resource competitiveness, and adapt better to environmental changes than the rare taxa (39, 57, 58). These bacteria may be abundant because their greater niche breadth allows them to develop better in a highly heterogeneous environment such as soil. Moreover, most of the N-sensitive bacterial species were rare taxa (Fig. 3), further suggesting higher sensitivity of the rare taxa to environmental perturbations, such as N addition. The N-sensitive bacterial species, such as members belonging to the *Acidobacteria* and *Proteobacteria*, dominated in the study forest, and their relative abundances were significantly influenced by N addition (Fig. S3). The results are consistent with previous studies showing that the phyla *Acidobacteria* and *Proteobacteria* are dominant in temperate forests, croplands, grasslands, and deserts and highly responsive to N input (14, 59, 60). The members of the *Acidobacteria* are acid-tolerant and oligotrophic and have the ability to degrade complex and recalcitrant carbon (61, 62). Meanwhile, the members of *Proteobacteria* are ubiquitous and copiotrophic, so they have fast growth rates under nutrient-rich conditions (63). Additionally, species belonging to the *Acidobacteria* and *Proteobacteria* are also found to be sensitive to other environmental changes, such as global warming, heavy metal contamination, and salinity gradient (64–66).

### Broader environmental acclimation of the abundant bacterial taxa.

We found that about 9% of soil bacterial species were assigned to the abundant taxa and were ubiquitous across all samples but that the rare taxa were unevenly distributed and mostly occurred in only a few samples (Table S1; Fig. S1). Bickel and Or (24) also revealed that common (abundant) bacterial species were prevalent across biomes on a global scale, but in their case only 0.4% of bacterial species were considered to be common, despite the same method being used to distinguish abundant from rare (24). The higher proportions (9%) of the abundant taxa in the present study might be attributed to the local scale, in which there would be a lower habitat diversity than across biomes. This would allow a greater proportion of the species to colonize all/most of the habitats. We also observed that the abundant bacteria exhibited broader environmental tolerance than the rare taxa, which is in line with previous studies of terrestrial ecosystems (34, 67, 68). One possible reason is that the abundant taxa have more individuals to disperse easily and could effectively access a broader array of resources than the rare taxa (42, 43). Additionally, the intrinsic genomic traits of abundant taxa enable their ubiquitous distributions, such as the positive relationships between abundance and gene occupancy (58, 69). Furthermore, stronger phylogenetic signals for environmental preferences were present in the abundant taxa than in the rare taxa, based on Blomberg’s *K* statistic. This suggests that closely related abundant species presented more similar ecological preferences across environmental gradients. Previous studies have implied that microbial responses to environmental disturbances were phylogenetically conserved, and the evolutionary history of their environmental acclimation could be predicted by phylogenetic signals that rely on ecological preferences (70, 71). Therefore, the stronger phylogenetic signals for the ecological preferences of the abundant taxa might suggest greater phylogenetic conservatism of their environmental acclimation in response to N addition. The findings mentioned above indicate that the abundant and rare bacteria harbor distinct adaptability to various environmental conditions under N addition in tropical forests, with potential broader ranges for the abundant taxa.

### Assembly processes governing the abundant and rare bacterial taxa under N addition.

Deciphering the relative influence of deterministic and stochastic processes governing soil microbial community assembly under N addition is crucial for revealing the mechanisms underlying the stability and acclimation of soil microbial communities in the face of future global change (18, 72). The mean NTI values of the overall and rare taxa were significantly greater than zero and higher than those of the abundant taxa in both experiments (Fig. 5), indicating that the rare taxa are more closely phylogenetically clustered than the abundant taxa. Previous studies have demonstrated that environmental filtering can result in phylogenetic clustering in bacterial communities (73, 74). Therefore, the rare taxa were more governed by deterministic processes than the abundant taxa in the present study. These results are consistent with previous studies that show that rare taxa are primarily influenced by deterministic processes in agricultural (43, 75), dryland (76), and wetland soils (34). However, they contradict the studies performed in oil-contaminated soils, rice paddy soils, and subtropical bays (39, 77, 78). Such inconsistencies might be due to the differences in geographic scales (47, 48) and/or the habitat conditions (49). Additionally, the broader environmental acclimation of the abundant taxa could further explain why they were less influenced by deterministic processes than the rare ones (25, 79). In contrast, the rare taxa have narrow niche breadths and limited dispersal ability due to their small population sizes, and as a result, their distributions are mainly driven by deterministic processes (23, 25). Moreover, the long-term high-N addition significantly decreased the mean NTI values of the abundant taxa, suggesting that N addition increased the stochasticity of the abundant taxa. It is possible N inputs would cause the early-abundant bacterial species to inhibit or promote late-arriving bacterial species, which is known as a priority effect (a stochastic process) (18, 41). Our findings, therefore, highlight that the relative importance of stochastic and deterministic processes in driving the assemblages of the abundant and rare taxa would be modified by the environmental changes triggered by N addition.

**FIG 6:**
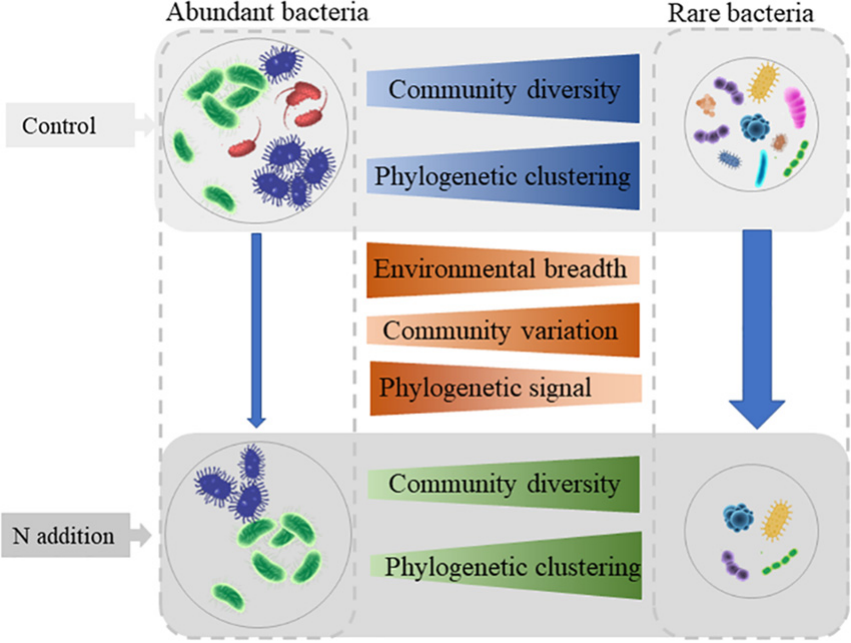
A conceptual paradigm showing responses of the abundant and rare bacterial communities to N additions in tropical forests. The arrows indicate the effects of N addition to bacterial diversity and community composition, and the size of the arrows indicates the strength of influence (stronger for the rare taxa and weaker for the abundant taxa). The blue trapezoids indicate the differences between abundant and rare taxa in control soils; the green ones show the differences between abundant and rare taxa in N addition soils; the orange ones represent the differences between abundant and rare taxa in both the control and N addition soils.

In summary, we provide a novel perspective on the responses of soil bacterial communities to anthropogenic N addition and propose a conceptual paradigm illustrating the effects of N addition on the abundant and rare bacterial taxa in tropical forest soils (Fig. 6). N additions significantly decreased the bacterial α-diversity and shifted the community composition toward copiotrophic bacteria, particularly in the long-term experiment. The abundant and rare soil bacteria responded differently to N addition, with more varied α-diversity and community structure of the rare taxa. Meanwhile, the abundant taxa showed broader environmental acclimation than the rare taxa, whereas the rare taxa were more closely clustered phylogenetically and, therefore, were more governed by deterministic processes than the abundant taxa. Together, these findings enhance our knowledge of the formation and maintenance of bacterial diversity and offer a way to better predict bacterial responses to the ongoing atmospheric nitrogen deposition in tropical forests.

## MATERIALS AND METHODS

### Site description and experimental design.

The present study was conducted in Dinghushan Biosphere Reserve (DHSBR) in the city of Zhaoqing, Guangdong province of south China (112°10′E, 23°10′N). The reserve covers approximately 1,155 ha with a monsoon climate. The mean annual precipitation from 2011 to 2020 was 1,414 mm, having a distinct seasonal pattern, with 70% of the rainfall concentrated in the period from April to September. The mean annual temperature from 2011 to 2020 was 22.4°C, with a monthly average temperature of 13.7 and 28.7°C in January and July, respectively (80). The total N deposition in the reserve was 49.8 kg N ha^−1 ^yr^−1^ in 2015 to 2016 (81). The study site is located in an evergreen broadleaf forest, and it has been regarded as N rich but P limited in previous studies (82). The main soil type is lateritic red soil (Hapli-Udic Ferralosols) formed from sandstone with a pH below 4.0 (2), and the dominant tree species are Castanopsis chinensis Hance, Machilus chinensis (Champ. ex Benth.) Hemsl., Schima superba Gardn. et Champ., Cryptocarya chinensis (Hance) Hemsl., Cryptocarya concinna Hance, and Randia canthioides Champion ex Bentham.

The short-term and long-term N addition experiments were set up in nearby tropical forest sites (~2 km apart) with similar plant community compositions and climatic conditions. Both of the experiments were laid out in a completely randomized block design. Specifically, the short-term experiment was established in October 2013 and received N additions from September 2014, with rates of 0, 35, 75, and 105 kg N ha^−1 ^yr^−1^ in control, low-N, medium-N, and high-N treatments, respectively. A total of 12 (3 replicates per treatment × 4 treatments) randomly distributed plots (15 m by 15 m per plot) were set up. N additions were carried out by spraying an ammonium nitrate (NH_4_NO_3_) solution on a monthly basis (30 L of solution over the year). The control plots received an equivalent volume of water only, which corresponded to an increase in precipitation of 1.6 mm per year. The long-term experiment was started in 2002, and N additions began in July 2003. Four N addition treatments with three replicates for each treatment were set up: control (without N), low N (50 kg N ha^−1 ^yr^−1^), medium N (100 kg N ha^−1 ^yr^−1^), and high N (150 kg N ha^−1 ^yr^−1^). The N was also added in the form of NH_4_NO_3_. The required NH_4_NO_3_ was dissolved in 20 L water and sprayed monthly below the canopy in each plot. There were 12 plots randomly distributed (10 m by 20 m) and surrounded by 10-m buffer strips between adjacent plots. Control plots received 20 L of water, which is equivalent to about 1.2 mm rainfall.

### Soil sampling and physicochemical properties.

Soil samples were collected from the surface (0 to 20 cm) of each plot in the two experiments in July 2015 and January 2016. Within each plot, samples were randomly taken using a soil auger (diameter, 5 cm) and mixed to form a composite sample for analysis. There were 48 soil samples in total. Soil samples were sieved through a 2-mm mesh in order to remove stones, visible roots, and plant residues. The sieved samples were divided into two subsamples. One subsample was used to determine soil properties, and the other was kept at −80°C for subsequent DNA extraction and molecular analyses.

Soil physicochemical properties, including soil water content (SWC), pH, ammonium N (NH_4_^+^-N), nitrate N (NO_3_^-^–N), total organic carbon (TOC), total N (TN), total phosphorus (TP), dissolved organic carbon (DOC) and dissolved organic N (DON), were measured as described previously (Table S4) (72).

### Molecular analyses.

Total DNA was extracted from soil samples using a PowerSoil DNA isolation kit (MoBio Laboratories, Inc., Carlsbad, CA, USA) according to the manufacturer’s instructions. DNA concentrations and quality were estimated with a NanoDrop instrument. The paired primers 515F/907R (5′-GTGCCAGCMGCCGCGGTAA-3′/5′-CCGTCAATTCMTTTRAGTTT-3′) were used to amplify the V4-V5 region of the bacterial 16S rRNA genes (83). The paired-end high-throughput sequencing of the16S rRNA gene was performed using the Illumina HiSeq platform (PE 250) by Magigene (Guangdong Magigene Biotechnology Co., Ltd., Guangzhou, China).

After sequencing, quality control and trimming of the paired-end sequencing reads were performed using Fastp (v0.14.1, https://github.com/OpenGene/fastp) and cutadapt (https://github.com/marcelm/cutadapt/) to remove reads containing more than 10% unknown nucleotides and less than 80% bases with quality (Q-value) of >20, the short sequences (<200 bp), adapters, primers, and poly bases (84, 85). The paired-end cleaned reads were merged as raw tags using USEARCH (v10.0.240, http://www.drive5.com/usearch/) with a minimal overlap of 16 bp and mismatch with 5 bp. After quality-filtering, the effective clean tags were denoised to produce ASVs following the unoise3 algorithm using default settings, and chimeras were removed (86). Representative sequences of each ASV were assigned against the SILVA database (release 132) for bacterial taxonomy. The ASVs affiliated with archaea, chloroplasts, mitochondria, and eukaryotes were excluded from downstream analysis. To avoid random effects during the identification of rare taxa, ASVs in the whole data sets that contained fewer than 10 reads were discarded. A randomly selected subset of 18,126 sequences from each sample was obtained to standardize the sequencing effort across samples. The abundant and rare taxa were classified based on thresholds set by minimizing cross-entropy using the autothresholdr package in R (24). This approach provides a nonbiased estimate of the classification threshold (24) and has been used to identify rare events (87). Detailed descriptions of the abundant and rare bacterial ASV data sets are presented in Table S1.

### Statistical analysis.

Unless otherwise specified, all statistical analyses were performed in the R environment (v4.0.4, http://www.r-project.org/). A paired-sample *t* test with Bonferroni correlation was performed to compare the bacterial α-diversity (richness, Shannon index, and Pielou’s evenness) of overall, abundant, and rare taxa at each N level between wet and dry seasons in the short- and long-term experiments. As the results showed that season had no significant effect on bacterial α-diversity, we considered the season as the replicates for downstream analysis (Tables S5 and S6). Therefore, one-way analysis of variance (ANOVA) with N level and block as factors followed by Tukey’s honestly significant different (HSD) test was performed to assess the differences of overall, abundant, and rare bacterial α-diversity and the relative abundance of major phyla among N-addition treatments in the short- and long-term experiments. These parameters were explored for normality (Shapiro-Wilk test) and homogeneity of variances (Bartlett test) prior to the one-way ANOVA analyses and were Box-Cox transformed if necessary. The Friedman rank sum test was performed when the data still did not meet the assumptions of normality and homoscedasticity after transformation. Between-class analysis (BCA) was carried out on ASV data to analyze the effects of N additions on the composition of the overall, abundant, and rare bacterial communities in each experiment, using the package ade4 in R (88). Monte Carlo tests were performed with 999 permutations to identify differences among treatments in overall, abundant, and rare taxa in the short- and long-term experiments. The results were visualized in ordination plots. Spearman’s rank correlation and simple Mantel tests were used to test the correlations of environmental variables and bacterial α-diversity, community composition, and the relative abundance of major phyla of overall, abundant, and rare taxa in each experiment.

To explore the most discriminant ASVs among N addition levels in the short- and long-term experiments, the nsASVs were screened by both the indicator species analysis using the indicspecies package (89) and likelihood ratio tests using the edgeR package (90) according to Hartman (91). The significant connections of nsASVs with the different N levels were visualized by bipartite networks using the Fruchterman-Reingold layout with 10^4^ permutations as implemented in the igraph package (92).

Threshold indicator taxon analysis (TITAN) was performed to evaluate the effects of physicochemical variables on environmental thresholds of the abundant and rare bacterial taxa using the TITAN2 package (93). To assess whether ecological traits could be predicted in phylogenetic levels, the strengths of the relationship between environmental preferences and bacterial phylogeny (phylogenetic signals) were examined for the abundant and rare bacterial taxa with Blomberg’s *K* analysis using the multiPhylosignal function in the picante package (94, 95).

The nearest taxon index (NTI) was used to determine the ecological processes (deterministic versus stochastic processes) that governed the bacterial community assembly. NTI was calculated using the null model taxa.labels (abundance.weighted = TRUE, null.model = taxa.labels, iterations = 1,000) with 999 randomizations across all samples in the ses.mntd function of the picante package (95). The mean NTI values from all communities were significantly above zero, indicating phylogenetic clustering, while the mean NTI values were significantly below zero, negative, denoting phylogenetic overdispersion. A larger absolute NTI value indicates greater effects of deterministic processes (96, 97). Differences in NTI values among N addition levels for overall, abundant, and rare taxa were compared by one-way ANOVA in the short- and long-term experiments.

### Data availability.

The raw sequence data for the 16S rRNA gene amplicons were deposited in the Sequence Read Archive (SRA) at the NCBI under accession no. PRJNA830431.
