# Supplementary material for: Distinct Responses of Abundant and Rare Soil Bacteria to Nitrogen Addition in Tropical Forest Soils
Source: Microbiol Spectr. 2023 Jan 9;11(1):e03003-22. doi: 10.1128/spectrum.03003-22 (PMC9927163; doi:10.1128/spectrum.03003-22)
Supplement: Supplemental file 1 — Supplemental material. Download spectrum.03003-22-s0001.pdf, PDF file, 1.3 MB [file spectrum.03003-22-s0001.pdf]

SUPPLEMENTARY MATERIAL

Distinct responses of abundant and rare soil bacteria to N addition in tropical forest soils

Jinhong He[a, #], Xiangping Tan[a, #], Yanxia Nie[a], Lei Ma[b], Juxiu Liu[a], Xiankai Lu[a], Jiangming Mo[a], Julie Leloup[c], Naoise Nunan[c, d, *], Qing Ye[a, e], Weijun Shen[f, *]

[a] Key Laboratory of Vegetation Restoration and Management of Degraded Ecosystems, South China Botanical Garden, Chinese Academy of Sciences, Guangzhou, China

[b] Key Laboratory of Geospatial Technology for Middle and Lower Yellow River Regions (Henan University), Ministry of Education, Kaifeng, China

[c] Institute of Ecology and Environmental Sciences - Paris, Sorbonne Université -CNRS-IRD-INRAE-Université Paris Cité-UPEC, 4 place Jussieu, 75005 Paris, France

[d] Department of Soil and Environment, Swedish University of Agricultural Sciences; P.O. Box 7014, SE-75007, Uppsala, Sweden.

[e] College of Life Sciences, Gannan Normal University, Ganzhou, China

[f] Guangxi Key Laboratory of Forest Ecology and Conservation, State Key Laboratory for Conservation and Utilization of Agro-bioresources, College of Forestry, Guangxi University, Nanning, Guangxi 530004, China

[#] These authors contributed equally.

*** Corresponding authors:**

Naoise Nunan

Institute of Ecology and Environmental Sciences - Paris, Sorbonne Université -CNRS-IRD-INRAE-Université Paris Cité-UPEC, 4 place Jussieu, 75005 Paris,

France

Email: naoise.nunan@cnrs.fr

Weijun Shen

College of Forestry, Guangxi University

100 Daxue Rd., Xixiangtang District

Nanning, Guangxi 530004, China

Tel/ Fax: (+86) 0771- 3271428

Email: shenweijun@gxu.edu.cn

Supplementary Table 1. A detailed description of abundant and rare ASVs in the short- and long-term experiments, respectively.

Category	Short-term				Long-term			
	ASVs		Sequences		ASVs		Sequences	
Abundant taxa	493	9.1%	298222	68.6%	493	8.9%	297044	68.3%
Rare taxa	4957	90.9%	136663	31.4%	5036	91.1%	137866	31.7%
Overall	5450	100.0%	434885	100.0%	5529	100.00%	434910	100.0%

Supplementary Table 2. Spearman’s correlation between bacterial α-diversity indices and soil properties

	Short-term									Long-term								
	Overall			Abundant			Rare			Overall			Abundant			Rare		
	Richness	Shannon	Pielou	Richness	Shannon	Pielou	Richness	Shannon	Pielou	Richness	Shannon	Pielou	Richness	Shannon	Pielou	Richness	Shannon	Pielou
SWC	0.13	0.18	0.27	-0.10	0.19	0.18	0.13	0.23	0.12	-0.23	-0.18	-0.10	0.29	-0.08	-0.12	-0.24	-0.25	-0.10
NH_4^+-N	-0.03	0.08	0.08	-0.14	0.06	0.10	-0.03	-0.09	-0.15	-0.19	-0.15	-0.10	-0.24	-0.36	-0.37	-0.19	**-0.47***	**-0.49***
NO_3^--N	-0.01	-0.08	-0.10	0.17	0.06	0.10	-0.01	0.07	0.25	-0.37	-0.19	-0.04	-0.39	-0.30	-0.27	-0.36	**-0.45***	-0.32
pH	-0.27	-0.29	-0.29	-0.07	**-0.42***	**-0.49***	-0.27	-0.32	-0.33	**0.62****	**0.47***	0.31	0.38	**0.42***	**0.43***	**0.62****	**0.67*****	**0.61****
TOC	0.18	0.33	**0.43***	-0.03	**0.46***	**0.51***	0.18	0.22	0.14	-0.36	-0.26	-0.17	0.02	-0.20	-0.23	-0.37	**-0.41***	-0.34
TN	0.27	0.26	0.32	-0.20	0.25	0.31	0.27	0.20	-0.03	-0.22	-0.23	-0.19	-0.15	-0.27	-0.29	-0.23	-0.34	-0.39
C/N	-0.09	0.11	0.16	0.24	0.25	0.23	-0.09	-0.02	0.07	-0.20	-0.08	-0.03	0.15	0.14	0.12	-0.20	-0.06	0.08
TP	0.07	-0.06	-0.08	-0.36	-0.15	-0.07	0.07	-0.02	-0.14	-0.29	-0.29	-0.23	0.00	-0.23	-0.26	-0.31	-0.34	-0.27
N/P	0.16	0.28	0.38	0.13	0.40	0.39	0.16	0.21	0.18	-0.09	-0.19	-0.20	-0.28	-0.19	-0.20	-0.10	-0.18	**-0.42***
C/P	0.04	0.25	0.35	0.27	**0.50***	**0.47***	0.04	0.18	0.26	-0.26	-0.14	-0.06	0.00	0.03	0.01	-0.26	-0.21	-0.25
DOC	-0.27	-0.29	-0.29	**-0.51***	-0.38	-0.34	-0.27	-0.13	0.12	-0.15	-0.11	-0.05	0.22	0.09	0.08	-0.15	-0.07	-0.15
DON	0.15	0.03	-0.03	-0.32	-0.02	0.06	0.15	0.17	0.14	-0.09	0.03	0.13	0.09	0.04	0.02	-0.10	-0.18	-0.18

Numbers in the table indicate the correlation coefficient (ρ). *** $p < 0.001$; ** $p < 0.01$; * $p < 0.05$; SWC, soil water content; NH_4^+-N, ammonium nitrogen; NO_3^--N, nitrate nitrogen; TOC, total organic carbon; TN, total nitrogen; C/N, total organic carbon/ total nitrogen; TP, total phosphorus; N/P, total nitrogen/ total phosphorus; C/P, total organic carbon/ total phosphorus; DOC, dissolved organic carbon; DON, dissolved organic nitrogen.

Supplementary Table 3. Simple Mantel tests for the correlations between environmental variables (Euclidean distance) and the bacterial community composition of the three taxa (Bray-Curtis distance) with 999 permutations in the short- and long-term experiments, respectively.

	Short-term						Long-term					
	Overall		Abundant		Rare		Overall		Abundant		Rare	
	ρ	*p*	ρ	*p*	ρ	*p*	ρ	*p*	ρ	*p*	ρ	*p*
SWC	0.08	0.20	0.07	0.22	0.07	0.25	0.00	0.48	-0.04	0.67	0.04	0.31
NH_4^+-N	0.08	0.20	0.04	0.31	0.07	0.24	0.03	0.33	0.00	0.45	0.05	0.25
NO_3^--N	-0.01	0.50	-0.05	0.73	-0.02	0.58	0.01	0.40	0.00	0.41	0.03	0.31
pH	0.08	0.20	0.07	0.22	0.04	0.38	**0.40**	**0.002**	**0.40**	**0.001**	**0.40**	**0.001**
TOC	0.03	0.36	0.06	0.24	0.01	0.46	0.08	0.21	0.04	0.34	0.12	0.09
TN	-0.17	0.97	-0.14	0.93	-0.20	0.96	-0.01	0.54	-0.02	0.56	0.03	0.30
C/N	0.02	0.42	0.06	0.27	-0.04	0.66	0.16	0.05	0.15	0.08	**0.17**	**0.038**
TP	0.11	0.09	0.10	0.12	0.12	0.10	-0.02	0.57	-0.04	0.64	0.02	0.37
N/P	0.02	0.42	0.05	0.29	-0.02	0.57	0.00	0.49	0.01	0.44	0.02	0.39
C/P	**0.23**	**0.009**	**0.27**	**0.004**	**0.17**	**0.038**	0.10	0.14	0.07	0.23	0.12	0.08
DOC	0.03	0.38	0.06	0.20	-0.01	0.54	0.02	0.34	-0.01	0.49	0.06	0.22
DON	-0.05	0.73	-0.03	0.63	-0.08	0.88	0.01	0.41	-0.01	0.54	0.04	0.26

ρ is the Spearman's rank correlation coefficient. Data in bold indicates significant correlations ($p < 0.05$); SWC, soil water content; NH_4^+-N, ammonium nitrogen; NO_3^--N, nitrate nitrogen; TOC, total organic carbon; TN, total nitrogen; C/N, total organic carbon/ total nitrogen; TP, total phosphorus; N/P, total nitrogen/ total phosphorus; C/P, total organic carbon/ total phosphorus; DOC, dissolved organic carbon; DON, dissolved organic nitrogen.

Supplementary Table 4. Variations of soil physiochemical properties (average ± standard error) under control, low-N, medium-N and high-N treatments in the short- and long-term experiments, respectively. Different lowercase letters indicate significant differences (p < 0.05) among different N-treatments in the short-term or long-term experiment, respectively.

Site	Treatment	SWC (%)	NH_4^+-N mg kg^{-1}	NO_3^--N mg kg^{-1}	pH	TOC %	TN %	C/N	TP %	N/P	C/P	DOC mg kg^{-1}	DON mg kg^{-1}
Short-term	Control	35.8±0.5	3.5±0.7	5.9±1.2^{b}	3.9±0.03	2.5±0.1	0.17±0.01	14.8±0.6	0.02	7.9±0.3	116.4±6.3	185.6±19.0	32.3±3.0
	Low-N	33.6±0.9	5.6±1.2	6.8±0.9^{b}	3.9±0.03	2.6±0.2	0.17±0.01	15.1±0.9	0.02	7.3±0.3	110.5±9.3	212.7±12.3	36.4±3.7
	Medium-N	34.9±1.2	5.0±0.6	8.2±0.5^{ab}	3.9±0.01	2.8±0.2	0.19±0.02	15.2±1.2	0.02	8.1±0.7	121.3±8.4	217.7±16.7	39.7±3.4
	High-N	33.7±0.5	3.5±0.4	9.6±0.8^{a}	3.9±0.03	2.6±0.1	0.17±0.01	15.1±0.4	0.02	8.1±0.2	123.1±4.1	191.2±13.8	34.9±3.4
Long-term	Control	31.8±1.3	3.8±1.3	5.9±1.3^{b}	3.9±0.03^{a}	2.5±0.1	0.17±0.02	15.3±0.9	0.02	8.4±0.4	126.6±2.5	177.9±17.3	35.2±5.2
	Low-N	33.3±1.7	5±1.2	7.9±1.5^{ab}	3.9±0.03^{ab}	2.5±0.3	0.19±0.02	13.5±0.9	0.02	8.9±0.4	118.9±8.1	172.4±17.3	38.3±4.6
	Medium-N	33.9±1.2	4.2±0.9	8.8±1.0^{ab}	3.8±0.04^{bc}	2.9±0.3	0.2±0.02	15.2±0.7	0.02	8.8±0.6	134.0±9.5	153.7±7.5	37.7±2.5
	High-N	32.9±1.4	6.9±0.9	12.3±0.9^{a}	3.7±0.03^{c}	2.7±0.2	0.19±0.02	14.6±0.5	0.02	8.9±0.4	128.6±3.9	159.0±4.7	40.5±3.6

soil water content; NH_4^+-N, ammonium nitrogen; NO_3^--N, nitrate nitrogen; TOC, total organic carbon; TN, total nitrogen; C/N, total organic carbon/ total nitrogen; TP, total phosphorus; N/P, total nitrogen/ total phosphorus; C/P, total organic carbon/ total phosphorus; DOC, dissolved organic carbon; DON, dissolved organic nitrogen.

Supplementary Table 5. Paired-sample t-test with bonferroni correlation showing the effects of dry and season on the α-diversity of bacterial community for the overall, abundant and rare taxa in each N addition level of the short-term experiment, respectively.

Level	Season	Richness						Shannon index						Pielou's evenness					
		Overall		Abundant		Rare		Overall		Abundant		Rare		Overall		Abundant		Rare	
		t	*p*	*t*	*p*	*t*	*p*	*t*	*p*	*t*	*p*	*t*	*p*	*t*	*p*	*t*	*p*	*t*	*p*
Control	Wet Dry	0.3	1	-0.3	1	0.4	1	0.4	1	-0.1	1	0.02	1	0.5	1	-0.1	1	-3.5	0.3
Low-N	Wet Dry	-1.0	1	0	1	-0.9	1	-0.1	1	-0.2	1	1.0	1	-1.1	1	-0.2	1	1.3	1
Medium-N	Wet Dry	1.8	0.9	-1.3	1	1.9	0.8	-0.4	1	-0.3	1	2.2	0.6	-8.2	0.1	-0.2	1	0.8	1
High-N	Wet Dry	2.3	0.6	-0.5	1	2.4	0.6	2.5	0.5	1.5	1	2.2	0.5	2.2	0.9	1.6	1	1.6	1

Supplementary Table 6. Paired-sample t-test with bonferroni correlation showing the effects of dry and season on the α-diversity of bacterial community for the overall, abundant and rare taxa in each N addition level of the long-term experiment, respectively.

Level	Season		Richness						Shannon index						Pielou's evenness					
			Overall		Abundant		Rare		Overall		Abundant		Rare		Overall		Abundant		Rare	
			t	*p*	*t*	*p*	*t*	*p*	*t*	*p*	*t*	*p*	*t*	*p*	*t*	*p*	*t*	*p*	*t*	*p*
Control	Wet	Dry	1.8	0.9	-1.4	1	1.8	0.8	1.9	0.8	4.3	0.2	3.2	0.3	1.9	0.8	6.3	0.1	-0.8	1
Low-N	Wet	Dry	-2.7	0.5	7	0.1	-3.2	0.3	2.1	0.7	4.3	0.2	-0.5	1	3.5	0.3	4.2	0.2	-0.1	1
Medium-N	Wet	Dry	-1.8	0.9	-0.5	1	-1.6	1	-1.1	1	-1.5	1	-1.9	0.8	-0.8	1	-1.1	1	-1.8	0.9
High-N	Wet	Dry	0.9	1	1.1	1	0.9	1	0.7	1	0.2	1	0.1	1	0.6	1	0.1	1	0.6	1

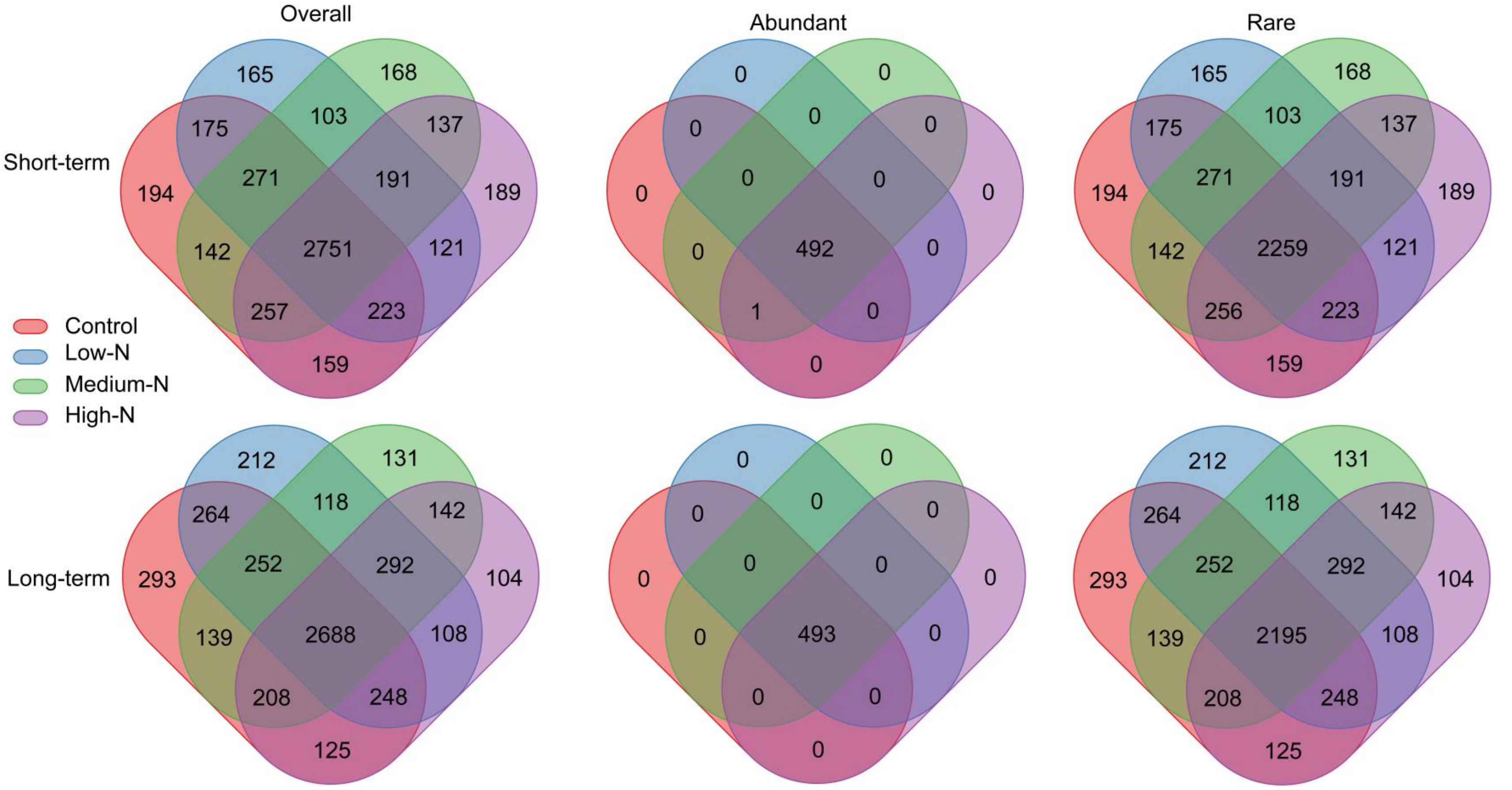

Supplementary Fig. 1 Venn diagram displaying the numbers of unique and shared ASVs in overall, abundant, and rare taxa across N treatments in the short- and long-term experiments, respectively.

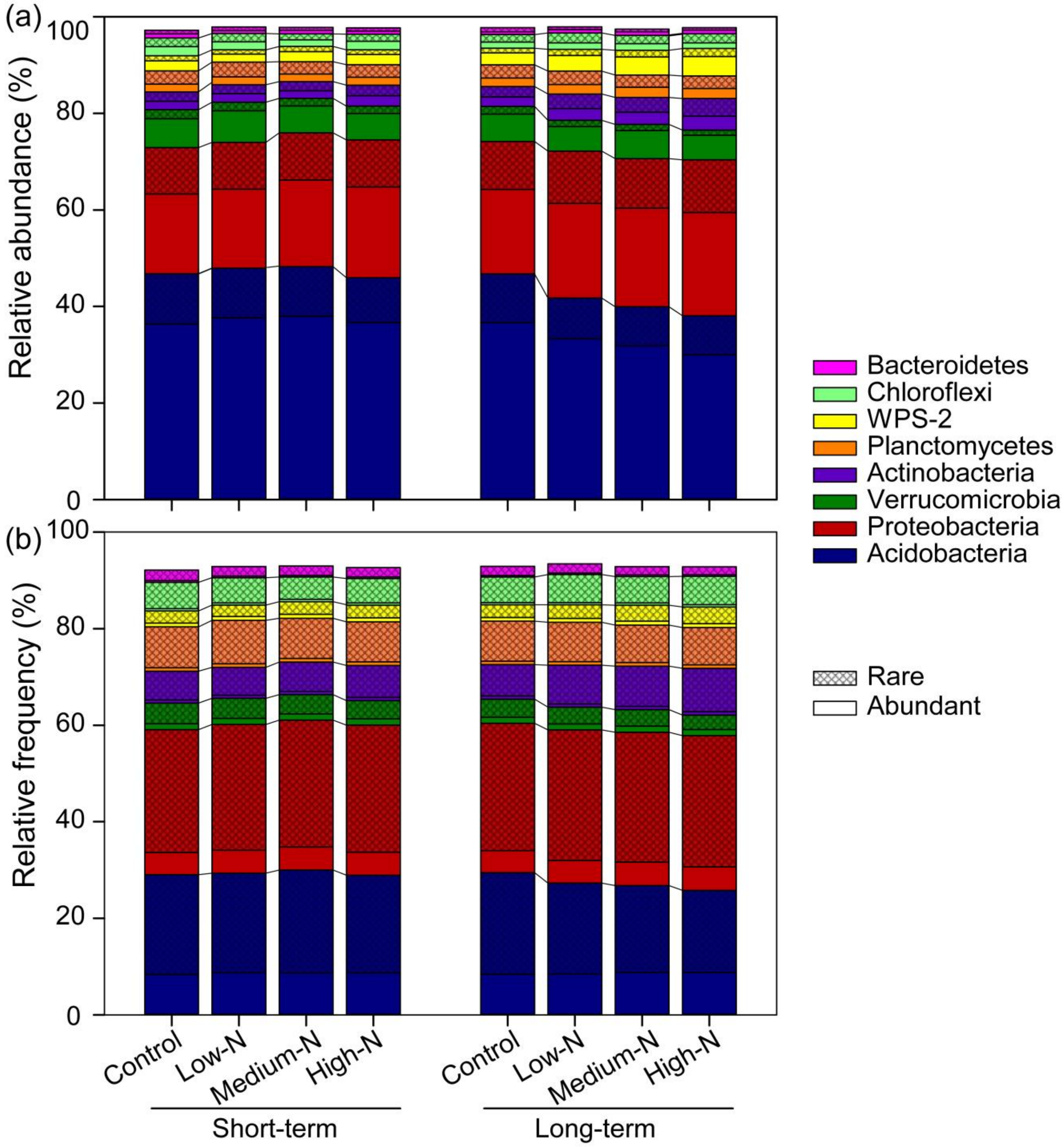

Supplementary Fig. 2 Relative abundance (a) and frequency (b) of major soil bacterial phyla across N addition levels in the short- and long-term experiments, respectively.

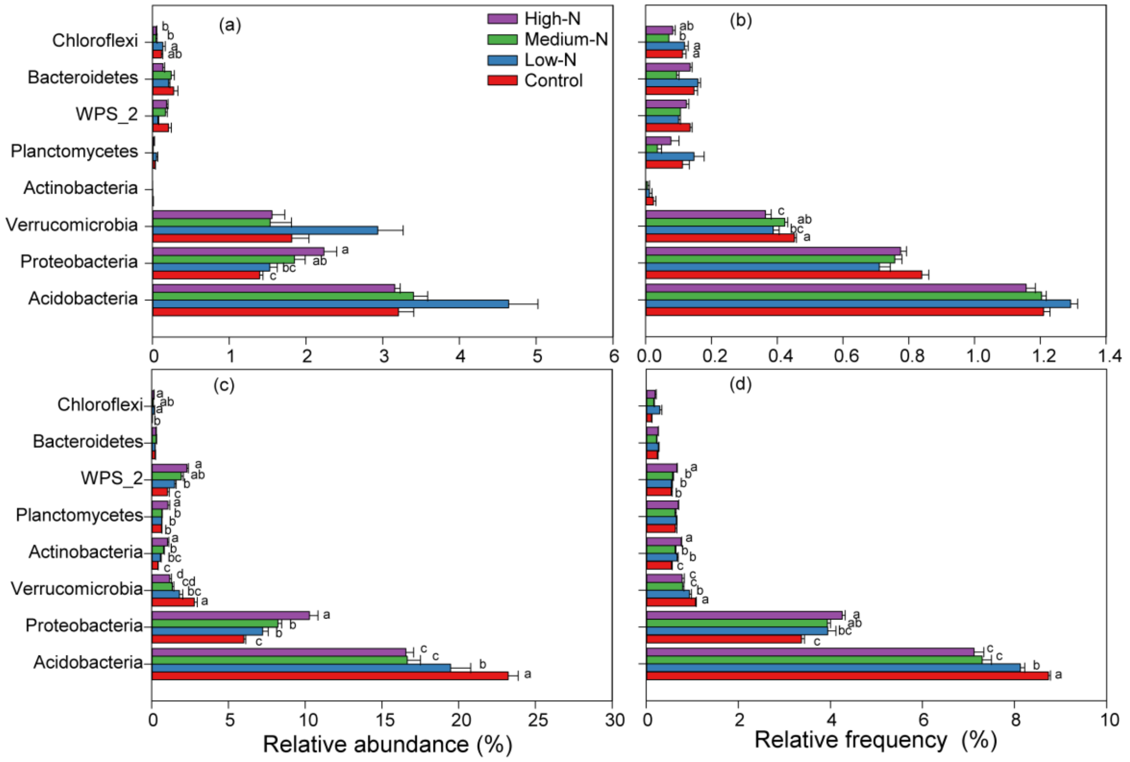

Supplementary Fig. 3 Variations of relative abundance and relative frequency of major sensitive phyla across N addition level in the short- (a, b) and long-term (c, d) experiments, respectively. The different lowercases denote the significant differences ($p < 0.05$) of major sensitive phyla among N addition levels.

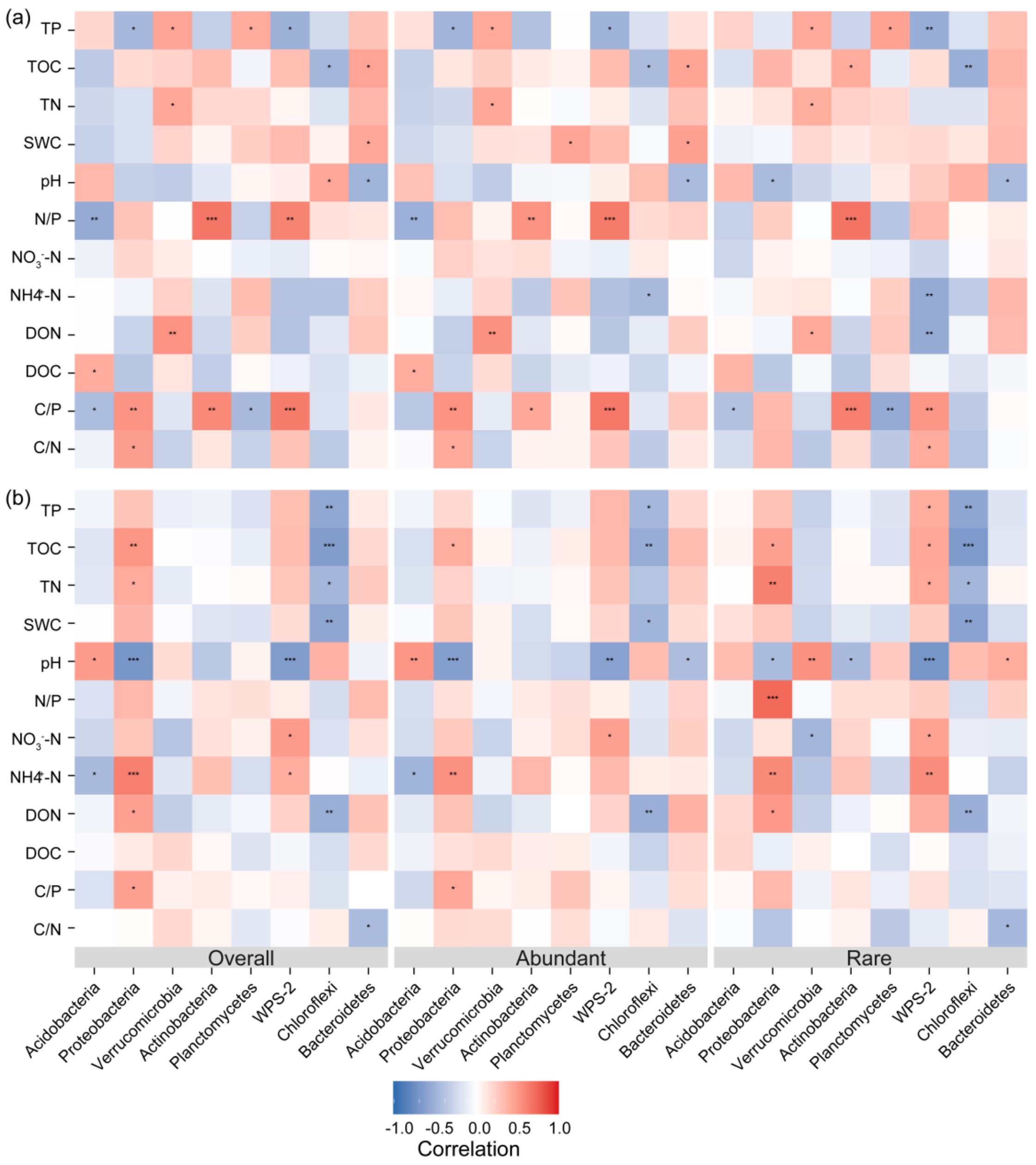

Supplementary Fig. 4 Heatmap of Spearman correlation on the relative abundance of major phyla and physicochemical parameters in the short- (a) and long-term (b) experiments, respectively. Negative and positive correlations are represented by blue color and red color. * indicates $0.01 < p \leq 0.05$, ** indicates $0.001 < p \leq 0.01$, *** indicates $p \leq 0.001$.
